# Beyond the synucleinopathies: alpha synuclein as a driving force in neurodegenerative comorbidities

**DOI:** 10.1186/s40035-019-0172-x

**Published:** 2019-09-04

**Authors:** Naomi P. Visanji, Anthony E. Lang, Gabor G. Kovacs

**Affiliations:** 1 Edmond J. Safra program in Parkinson’s disease and the Morton and Gloria Shulman Movement disorders clinic, Toronto Western Hospital, Toronto, Ontario Canada; 2 Department of Laboratory Medicine and Pathobiology and Tanz Centre for Research in Neurodegenerative Disease, University of Toronto, Toronto, Ontario Canada; 3 Laboratory Medicine Program & Krembil Brain Institute, University Health Network, Toronto, Ontario Canada

**Keywords:** Amyloid-β, Alpha-synuclein, Alzheimer’s disease, Comorbidity, Dementia with Lewy bodies, Multiple system atrophy, Neurodegeneration, Parkinson’s disease, Prion protein, Progressive supranuclear palsy, Proteinopathy, Tau, TDP-43

## Abstract

The fundamental role that alpha-synuclein (aSyn) plays in the pathogenesis of neurodegenerative synucleinopathies, including Parkinson’s disease, dementia with Lewy bodies, and multiple system atrophy, is a well-accepted fact. A wealth of experimental evidence has linked this relatively small but ubiquitously expressed protein to a plethora of cytopathologic mechanisms and suggests that aSyn may be capable of seeding the progressive spread of synucleinopathy throughout the brain. Beyond the synucleinopathies, the abnormal deposition of aSyn is frequently seen in a variety of other neurodegenerative proteinopathies including Alzheimer’s disease. In spite of the fact that the frequency of concomitant aSyn pathology in these disorders is such that it can be considered the rule rather than the exception, the potential role that aSyn may have in these disorders has received relatively little attention.

In this article we postulate that aSyn may in fact be a key protein in driving the pathogenic processes in neurodegenerative comorbidities. In addition to reviewing the frequency of concomitant deposition of aSyn in the neurodegenerative proteinopathies, we also consider our current understanding of the interaction of aSyn with other neurodegenerative disease-associated proteins, including tau, TDP-43, amyloid-β and prion protein, in the context of neuropathologic studies describing the anatomical sites of potential concomitant pathology. We conclude that a growing body of evidence, encompassing neuropathology studies in human brain, animal models of concomitant proteinopathies and studies employing sophisticated methods of probing protein-protein interaction, cumulatively suggest that aSyn is well positioned to exert a strong influence on the pathogenesis of the neurodegenerative comorbidities.

We hope to stimulate research in this emerging field and consider that future studies exploring the contribution of aSyn to the pathogenic processes in neurodegenerative comorbidities may provide critical information pertaining to diagnosis and the development of vital disease modifying treatments for these devastating diseases.

## Background

**Fig. 1:**
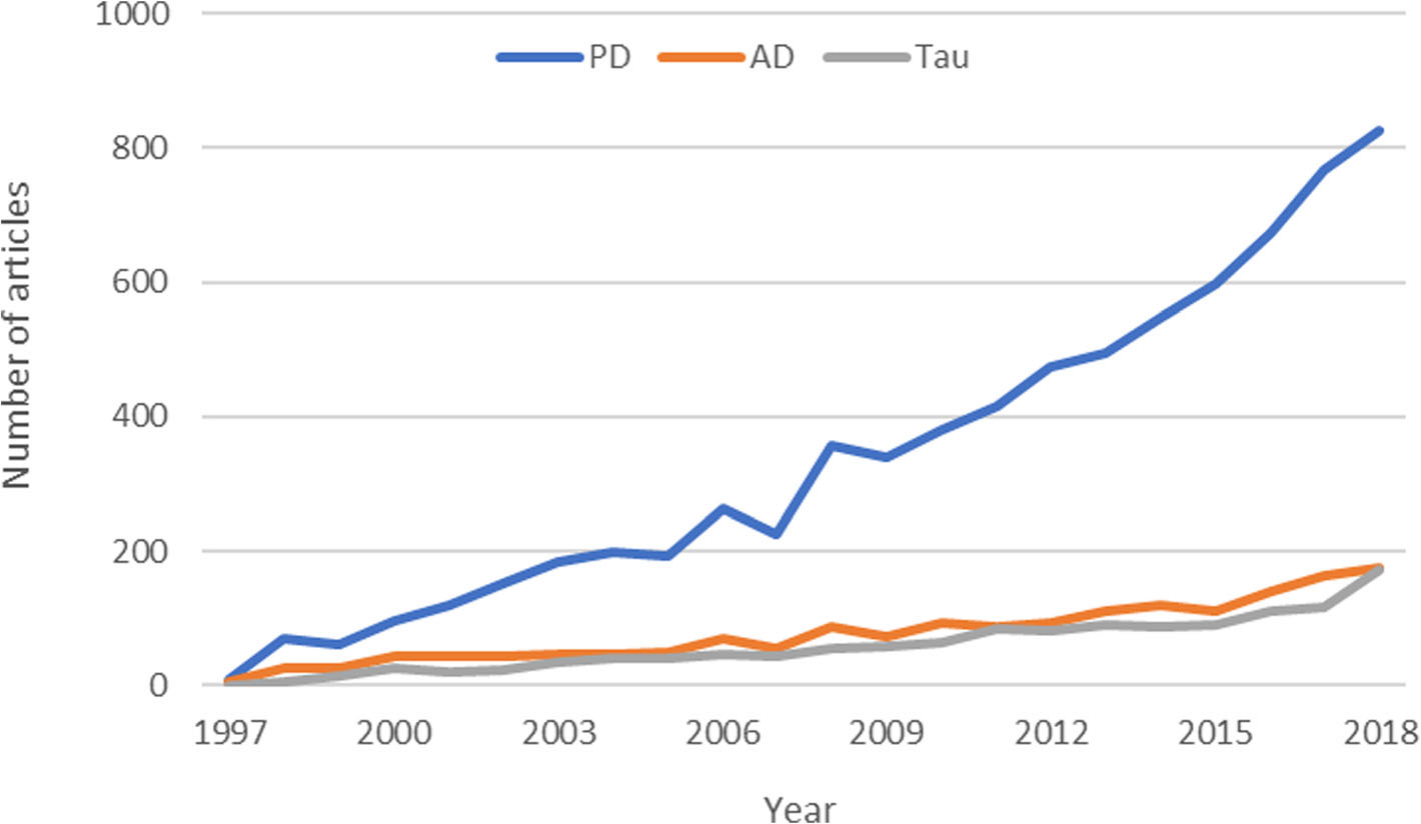
The number of articles in Pubmed by year containing the words alpha synuclein and either Parkinson’s disease, Alzheimer’s disease or tau from 1997 to 2018 ((alpha synuclein) AND Parkinson’s disease/Alzheimer’s disease/tau) AND (“1997”[Date - Publication]: “2018”[Date - Publication]) in the title

Alpha synuclein (aSyn) is a 14 kDa protein ubiquitously expressed in the presynaptic terminals of the brain, where it has been estimated to account for up to 1% of all cytosolic proteins [1, 2]. Since the discovery in 1997 that a mutation in *SNCA,* the gene that encodes aSyn, is linked to an autosomal dominant early-onset form of Parkinson’s disease (PD) [3] there has been an explosion of studies demonstrating the involvement of aSyn as a critical element of PD pathogenesis (Fig. 1). This more than of 20 years of research has yielded a wealth of evidence demonstrating that aggregated aSyn is a key feature of the neuropathology of PD and is heavily implicated in the neurodegenerative process in PD (reviewed in [4]). Some forms of aSyn aggregates can be neurotoxic and have been linked with a variety of deleterious effects in neurons including downregulation of mitochondrial complex1 activity [5], endoplasmic reticulum stress [6], neuroinflammation [7], disrupted cell membrane integrity [8] as well as inhibition of the ubiquitin proteasome system and impairment of the autophagy lysosomal pathway, which may in turn result in decreased degradation of aberrant aSyn, fueling a vicious neurotoxic cycle [9]. In addition, much recent attention has been paid to the observation that aSyn fibrils are seeds capable of inducing further aggregation of physiologic aSyn in a wide variety of model systems [10]. Collectively these findings have positioned aSyn at the epicenter PD research over the last 20 years. However, we are becoming increasingly aware that aSyn may also play a role in the pathogenesis of several other neurodegenerative proteinopathies.

There are several diseases in which pathologic aggregates of aSyn are a defining feature, collectively termed synucleinopathies. Primary synucleinopathies include Lewy body disorders, such as dementia with Lewy bodies (DLB), Parkinson’s disease (PD) PD with dementia (PDD) and pure autonomic failure (PAF). These are characterized by the predominance of intraneuronal cytoplasmic and neuritic deposits (Lewy bodies and Lewy neurites). The classification of these disorders is based on the clinical presentation and spatiotemporal development of aberrant aSyn pathology [11]. A further disorder, multiple system atrophy (MSA) is dominated by glial cytoplasmic inclusions (Papp-Lantos bodies). In addition to these primary synucleinopathies, deposition of aSyn is also commonly observed in the brains of individuals with other primary diagnoses (reviewed in [12, 13]). Aberrant accumulation of aSyn is frequently observed in brains with abnormal deposition of Tau, transactive response DNA binding protein 43 kDa (TDP-43), amyloid-β (Aβ) or prion protein. The frequency of these pathologic comorbidities is so common that their presence is the rule rather than the exception in neurodegenerative diseases. However, this frequency of co-occurrence is not reflected in published research, which has largely ignored this phenomenon. As illustrated in Fig. 1, although there are a large number of articles citing both aSyn and PD in the title, the number of articles citing aSyn in combination with either AD or tau in the title is disproportionately low.

Currently the pathological classification of neurodegenerative diseases is based on the predominant proteinopathy [11]. However, ignoring the existence of these comorbid proteinopathies that frequently exist simultaneously in the same brain likely impedes our understanding of disease pathogenesis, precludes the accurate early diagnosis of neurodegenerative proteinopathies and stratification of patients for clinical trials and jeopardizes the development of much needed disease modifying therapies. Here we review both the frequency of aSyn deposition in different neurodegenerative conditions and the available experimental studies on interaction of aSyn with other proteins associated with neurodegenerative disease with a focus on evidence suggesting aSyn may be a key protein in driving the neurodegenerative processes in these conditions. For clarity, it is our opinion that referring to the concomitant deposition of aSyn in diseases not classified as synucleinopathies as a “co-pathology” may infer a lesser importance of aSyn compared to the other abnormally deposited protein. We believe that, as the relative contribution of each aberrantly deposited protein to the neurodegenerative process is presently unknown, the term “concomitant pathology” is more appropriate. Concomitant is defined as “naturally accompanying or associated” reflecting both the frequency of this observation whilst remaining unbiased as to the relative importance of each aberrantly deposited protein to the neurodegenerative process as a whole.

## Main text

### Mechanisms of aSyn aggregation and propagation

Prior to considering any role aSyn may have in driving neuropathologic comorbidities it is important to appreciate the mechanisms by which aSyn has been shown to propagate synucleinopathy alone. This topic has been the focus of an impressive amount of research over the last decade inspired initially by the observation that aSyn pathology is evident in embryonic dopamine neurons grafted into the brains of human PD patients, leading to the hypothesis that aSyn may be capable of propagating synucleinopathy in a prion-like fashion [14]. There is now a wealth of experimental evidence ranging from studies in cultured cells to animal models demonstrating that aSyn seeds are capable of being transmitted from neuron to neuron and incorporating the aSyn of the host neuron into misfolded aggregates, leading to neuronal dysfunction and ultimately cell death (recently reviewed in [15]. Several studies interrogating the ability of aSyn to seed pathology in recipient cells or tissues have exploited preformed fibrils (PFFs) of misfolded aSyn generated by sonicating β-sheet rich fibrils of recombinant aSyn [16]. PFFs are rapidly taken up by numerous cell lines in culture, including primary dopamine neurons, whereby they induce the formation of aSyn aggregates bearing several similarities to Lewy bodies, including a high degree of phosphorylation at serine 129, polyubiquitination and coexpression of p62 [16–19]. Similarly, inoculation of both wildtype and transgenic animals with PFFs results in the development of widespread synucleinopathy throughout synaptically connected networks, neurodegeneration and behavioural deficits (reviewed in [15, 20]. Other studies have demonstrated similar effects utilizing aSyn derived from human brain tissue from individuals with synucleinopathies [17, 21]. Identifying the mechanisms for uptake (putatively receptor mediated), processing (predominantly lysosomal) and release of these toxic aSyn seeds is the topic of ongoing research efforts (reviewed in [15]), as well as the possible contribution of different conformational strains of aSyn to the different pathological inclusions observed among the synucleinopathies. Thus, aSyn extracted from glial cytoplasmic inclusions has been shown to have a different proteolytic profile and a much more potent biological activity than that of Lewy body derived aSyn [21]. There are obvious therapeutic implications of this aspect of aSyn biology with several efforts underway to prevent aSyn spreading synucleinopathy throughout the brain, including the recent initiation of clinical trials of antibodies directed at aSyn. Certainly a better understanding of the mechanisms of release, uptake and trafficking of internalized aSyn should help provide several novel targets with disease modifying potential in PD and related synucleinopathies in the near future.

### aSyn and comorbidity: a critical overview of neuropathological aspects

The association of synucleinopathy with different neurodegenerative conditions can be discussed from two perspectives. First, when aSyn appears as comorbidity (i.e., concurrent presence of brain disease with overlapping pathogenic aspects [13]) in diverse neurodegenerative conditions, and second when other proteinopathy comorbidities are observed in primary synucleinopathies. This is a somewhat arbitrary grouping as in cases with complex constellations of proteinopathies, it can be difficult to elucidate which is the predominant feature. In spite of the fact that emerging research is focusing on the description of concomitant pathologies, comparison between different studies will likely be hampered by the current lack of consensus regarding harmonization of nomenclature and evaluation strategies. There are several layers of complexity that must be kept in mind when evaluating the role of aSyn in proteinopathic comorbidities, thus simply referring to the presence of Lewy body pathology as a concomitant pathology might be insufficient for the reasons described below [13].

First, the classification of disorders with Lewy bodies awaits further crystallization. Diseases with Lewy bodies are grouped based on the early clinical presentation (movement disorder vs cognitive decline) [11]. However, although cluster analysis suggests that there are several distinct subtypes of PD [22], and several studies raise the concept that DLB might be distinct from PD and PDD [23–25], there are currently no known biochemical or morphological features of aSyn pathologies allowing a clear division of subtypes of Lewy body disorders. Importantly, the anatomical distribution of synucleinopathy and the concomitant presence of further protein deposits (e.g., Aβ and tau-positive neurofibrillary tangles) might be an important aspect for distinguishing clinically different Lewy body disorders [23, 25]. Finally, there are conditions, detectable only by neuropathologic study, when Lewy bodies accumulate solely in the amygdala or the olfactory bulb or lower brainstem without any clinical manifestation (i.e, incidental Lewy body disease), or only in peripheral organs [11, 26]. Currently, two neuropathological approaches are used to describe Lewy-body related pathology. Braak staging of Lewy body pathology, delineates the sequential involvement of brain regions starting in the medulla oblongata (stage 1), pons (stage 2), mesencephalon, in particular the substantia nigra (stage 3), limbic areas (stage 4), and finally reaching neocortical areas (stages 5 and 6) [27]. Intriguingly, not all cases strictly follow the sequential distribution described by Braak staging [28]. A second set of criteria, which originates from the classification of Kosaka [29], are implemented in the diagnosis of DLB as either brainstem, limbic or neocortical types [30]. Since these approaches do not recognize the early or pure involvement of the olfactory bulb and various predominances of aSyn deposition, this was included in further strategy, the so called unified staging system for Lewy body disorders. This suggests classification of cases into one of the following stages: I. Olfactory Bulb Only; IIa Brainstem Predominant; IIb Limbic Predominant; III Brainstem and Limbic; IV Neocortical [31].

Second, historical studies focused exclusively on the presence of Lewy bodies and their clinicopathological relevance. However, the spectrum of aSyn accumulations in Lewy body disorders is much broader than the mere presence of Lewy bodies and involves depositions in synapses, neurites, astrocytes, and oligodendrocytes [11, 32–34]. In addition, the use of modern techniques, such as the proximity ligation assay, has revealed further pathological alterations in neurons in Lewy body disorders [35]. Unfortunately, documentation of most of these aspects is lacking in the majority of existing clinicopathological studies, many of which also employed different antibodies for immunostaining, altogether jeopardizing our understanding of the role of aSyn pathology in diverse neurodegenerative conditions. These limitations call for a harmonized approach involving the evaluation of several anatomical regions using novel antibodies with standardized immunohistochemical, and other methods and a consensus description of anatomical patterns of different cellular or synaptic aSyn deposits to enable the comparison of different cohorts to enhance our understanding of the full spectrum of aSyn pathology in synucleinopathies.

The clinical subtypes associated with MSA pathology [36] cannot be clearly translated into biochemical or morphological differences. For the diagnosis of MSA the presence of oligodendroglial inclusions/glial cytoplasmic inclusions (CGIs) (Papp-Lantos bodies) is sufficient [37], however, neuronal aSyn pathology and further pathologies, described recently by sophisticated methods [38], should be also considered. The distribution of glial inclusions might follow either a striatonigral or olivopontocerebellar predominance or even be associated with frontotemporal lobar degeneration (FTLD) and prominent neuronal aSyn accumulation in the medial temporal lobe [39, 40]. Although incidental MSA cases are described [41], MSA is usually not sought in the diagnostic screening for concomitant pathologies since early steps of the disease might involve only the cerebellum [42]. Indeed, an aging study showed that by immunostaining several anatomical regions asymptomatic MSA cases could be identified in elderly communities [43].

Despite the relative frequency of concomitant neurodegenerative disorders, pure forms of proteinopathies are still apparent using current diagnostic methods. This supports the current protein-based molecular classification [13]. The interpretation of different studies is complicated by the fact that abnormally deposited proteins exist in different phases of aggregation or fibrillization, and in different phases or stages of sequential involvement of anatomical brain areas, which in turn are influenced by genetic variations, age and sex effects and multimorbidities including systemic and vascular disorders [13]. Furthermore, TDP-43 pathology has only recently been added to the spectrum of concomitant proteinopathies and only a very few studies report its frequency. In addition, tau pathology is perceived mostly as neurofibrillary tangles related to AD, thus, subcortical and astroglial tau pathology or even primary age-related tauopathy (PART) or the limbic tauopathy argyrophilic grain disease (AGD) is considerably underappreciated in most studies. These caveats have led to a wide range of reported frequencies of neurodegenerative comorbidities depending on diverse case collection and neuropathological methodological strategies, which we have summarised below.

TDP-43 pathology is clearly more frequent in Lewy body disorders (generally around 20%) [44], while less frequent in MSA (4–7%) [44, 45]. Further studies indicate that TDP-43 deposition in DLB (33.3%) is less frequent than in mixed AD/DLB cases (52.6%), or in AD (73.9%); but more frequent then in aged controls (17.9%) [46]. A recent study reported more extensive Lewy body distribution correlating with more frequent Aβ deposition in brainstem (50%), limbic (57%) and neocortical forms (80%) of Lewy body disorders or TDP-43 deposition (0, 16 and 22%, respectively) [44]. When comparing the clinical phenotypes, PDD and DLB are associated with more frequent AD-related pathology and TDP-43 proteinopathy than PD, but a similar frequency of AGD, and lower prevalence of PART [47]. These changes are associated with different loads of Aβ and tau pathologies in diverse anatomical regions [25]. Importantly, in MSA TDP-43 immunoreactivities comprise subpial astrocytic inclusions and glial cytoplasmic inclusions in addition to neuronal inclusions, dystrophic neurites, and perivascular inclusions [45]. Furthermore, concomitant Lewy bodies have been described in up to 10% of MSA cases [48]. Regarding AD-related pathology, intermediate to high levels of AD neuropathological changes have been described in ~ 8%, and PART in approximately 40% of MSA cases [44]. Any type of ageing-related tau astrogliopathy (ARTAG) is detected in up to 56% of synucleinopathy cases, specifically grey matter ARTAG has been reported in 36.8% of Lewy body disorders and 17.1% of MSA cases [49]. In a small group of neuropathological controls (i.e. lack of any neurodegenerative disease entity including PART) any type of ARTAG was observed in 28.6% and grey matter ARTAG was lacking (0%) [49].

**Fig. 2:**
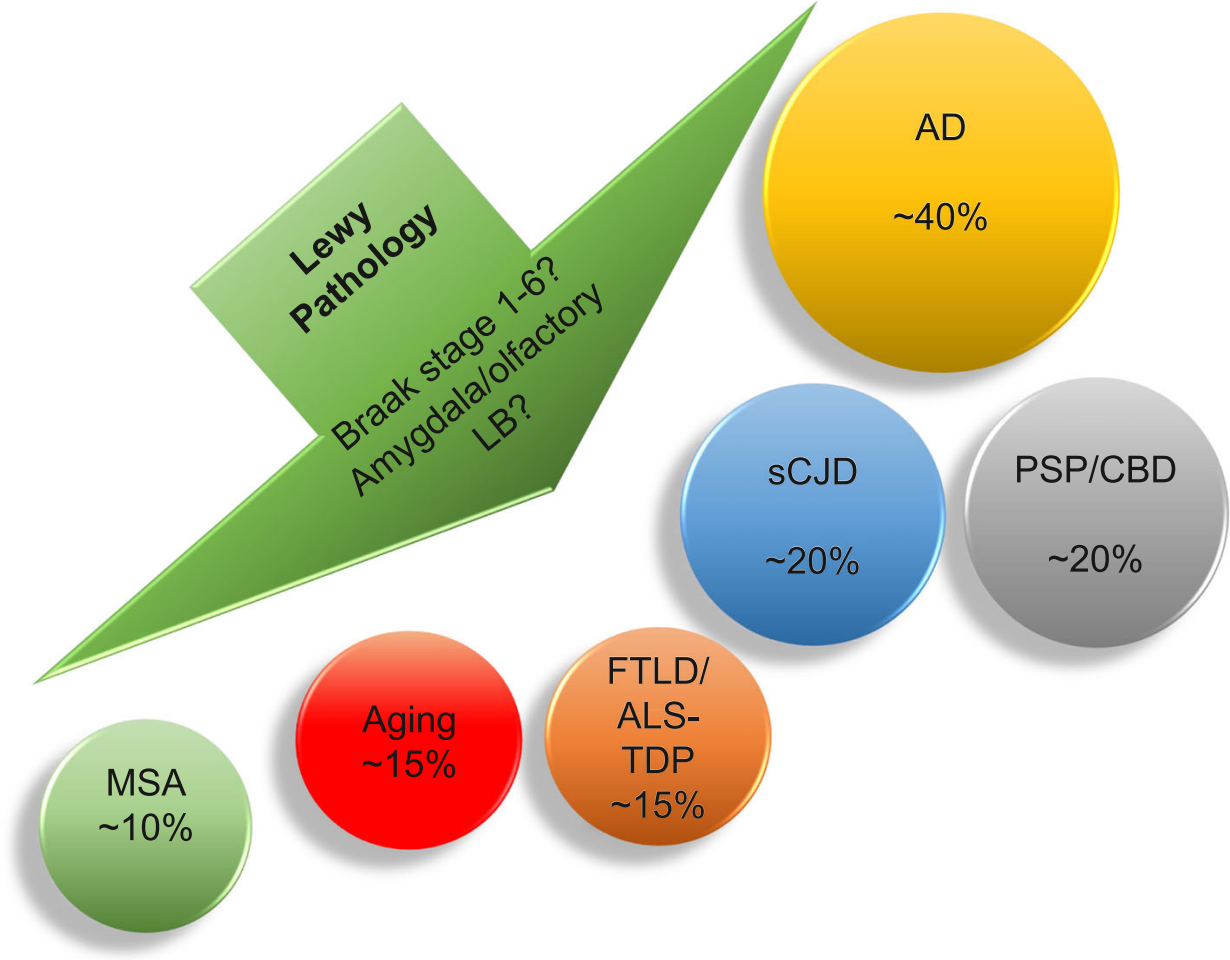
Approximate frequency of Lewy pathology reported in other neurodegenerative proteinopathies and in the unimpaired aged population. These estimates do not capture the amount of Lewy pathology present or anatomical distribution of Lewy pathology in relation to the primary pathology, but instead reflect the reporting of the presence of any amount of Lewy pathology in any brain region. Lewy body (LB); multiple system atrophy (MSA); unimpaired aging (Ua), frontotemporal lobe dementia (FTLD), amyotrophic lateral sclerosis (ALS), sporadic Creuzfeldt-Jakob disease (sCJD); Alzheimer’s disease (AD); progressive supranuclear palsy (PSP); corticobasal degeneration (CBD)

On the other hand, Lewy body pathology is reported in a wide range of neurodegenerative disorders and in unimpaired aging (Fig. 2). Studies in ageing cohorts, irrespective whether the individuals showed neurological symptoms or not, have typically focused on the presence of Lewy bodies related to AD-related pathological change and have reported frequencies ranging from 6 to 39% [12]. Studies employing various methods have suggested a frequency of concomitant Lewy bodies in ~ 20% in CBD and PSP, which is thought to be only slightly higher than that in the general aged population [44, 50, 51]. A somewhat lower prevalence of concomitant Lewy body pathology has been reported in FTLD-TDP and ALS-TDP cases (11–15%) [44] and in sporadic Creutzfeldt-Jakob disease (prion disease) (9–23%) [52, 53]. In comparison the prevalence of Lewy pathology in unimpaired aging has been reported as ~ 13% in a single study [54].

### Experimental studies on the interaction of aSyn with other neurodegenerative disease-associated proteins

1. *Tau*

Experimental studies suggest that aSyn and tau, including oligomeric forms, may directly interact with each other, furthermore, co-deposition of both proteins in Lewy bodies, Lewy neurites and tangles has been shown using antibodies against different epitopes of tau and aSyn in postmortem tissues (reviewed in [55]). The latter depends on the anatomical location and disease type exemplified by the relative paucity of co-localization in the substantia nigra in concomitant progressive supranuclear palsy and Lewy body pathology, whereas it is more likely to detect co-localization in the amygdala in concomitant AD and Lewy body pathology [56–58]. Whereas aSyn has a tendency to self-aggregate [59], the aggregation of tau relies on a variety of cofactors, many of which likely remain to be identified [60]. A seminal study by Giasson and colleagues implicated aSyn as a possible cofactor in the aggregation of tau [61]. Using recombinant aSyn and tau these authors showed a synergistic relationship whereby one protein can act to cross-seed the aggregation of the other in vitro*.* More recent studies in cultured cells, whilst supporting the ability of recombinant aSyn fibrils to induce aggregation of tau, however, have cast some doubt on the reciprocal ability of tau fibrils to cross-seed the aggregation of aSyn [62]. These discrepancies may reflect the disparate techniques used to determine interaction between these two proteins. Indeed, one study using Fluorescence Resonance Energy Transfer (FRET) microscopy, failed to show any cross-seeding between aSyn and tau [63]. This method together with proximity ligation assay also failed to provide clear evidence on the close proximity allowing direct interaction of tau and aSyn in human brain tissue in spite of rare co-localization in inclusion bodies in the amygdala [56]. Alternatively, these discrepancies may relate to variability in the capability of different conformational strains, or different antibodies raised against distinct biochemical modifications of these proteins in human studies, a further source of variability between studies, to interact with each other, as previously demonstrated by Guo and colleagues [64].

The observation that individuals from the Contursi kindred, who express the aggregation prone A53T mutation in aSyn, also have tau filamentous amyloid inclusions, as do mice that overexpress A53T or E46K mutant aSyn, provides evidence that mutant aSyn can drive aggregation of tau in vivo [61, 65, 66]. Furthermore, affinity chromatography in human brain lysates has revealed a direct interaction between the C terminus of wildtype aSyn and the microtubule binding domain of tau [67]. In addition, mice injected with recombinant preformed fibrils (PFFs) of wildtype aSyn develop not only a widespread synucleinopathy but also develop tau deposits, further supporting a synergistic relationship between aSyn and tau aggregation in vivo [68].

Interestingly, in an experimental setting there is little evidence of overlap between tau and aSyn deposits in either cultured cells or in mice exposed to aSyn PFFs [62, 69]. Furthermore, as tauopathy is frequently found in the absence of synucleinopathy in human brain [70], although evidence suggests aSyn may well be a co-factor able to drive the aggregation of tau, many further studies should be undertaken to determine the magnitude of any such effect in human comorbid proteinopathies.

2) *TDP-43*

TDP-43 is frequently associated with tau and Aβ, yet to date only two recent studies, from the same group, have addressed the interaction between aSyn and TDP-43 at an experimental level. SHSY5Y cells, double transfected with both aSyn and a mutant TDP-43 lacking the nuclear localization signal (TDP-43dNLS) developed abundant aggregates of phosphorylated TDP-43 upon exposure to aSyn PFFs [62]. These aggregates were not formed in cells expressing wildtype TDP-43, suggesting that the predominantly cytosolic TDP-43dNLS is more susceptible to the aggregation catalysing effects of aSyn PFFs than the predominantly nuclear wildtype TDP-43. In a second in vivo study, wildtype mice inoculated with intracerebral aSyn PFFs developed a synucleinopathy as well as abnormal dot-like TDP-43 deposits [68]. Similar to the distribution of PFF-induced synucleinopathy and tau deposition, inclusions of aSyn and TDP-43 were only partly colocalized in cells exposed to aSyn PFFs and rarely colocalized in mice exposed to aSyn PFFs calling into question the significance of any synergistic effect of aSyn on TDP-43 aggregation in disease [62, 68]. In human tissue rare co-localizations are observed for TDP-43 in amygdala Lewy bodies or aSyn in amygdala TDP-43 inclusions [56], while in MSA–related oligodendroglial aSyn inclusions occasionally TDP-43 immunoreactivity can be observed [45].

3) *Amyloid-β*

In 1993 Ueda and colleagues reported a study on an unrecognized component of amyloid in AD and tentatively named this 35-amino acid peptide NAC (non-Aβ component of AD amyloid) and its precursor NACP [71], which soon was defined as aSyn [1, 2]. Later it was shown that the amyloid core of Aβ plaques itself lacks aSyn deposition [72] but the protein is seen in the dystrophic neurites of plaques. These observations in human tissue sparked interest in examining these two proteins as potential interactors.

Indeed, experimental studies provide a wealth of evidence thought to support the binding and coaggregation of Aβ and aSyn. In vitro, Aβ can co-seed the aggregation of aSyn in both cell free and cell-based systems [73–78] and recombinant Aβ can induce phosphorylation of aSyn at ser 129 (aSyn-ser129P) [79]. In animal models, there is conflicting evidence regarding the interaction between aSyn and Aβ. Thus, the combined expression of human Aβ and aSyn has been shown to increase the deposition of intraneuronal fibrillar aSyn and accelerate the development of both motor and cognitive dysfunction in double transgenic mice [73]. Similarly in a mouse model of DLB-AD, that simultaneously expresses PS1(M146 V), APP(Swe), tau(P301L) and aSyn(A53T), a substantial increase in deposition of Aβ, tau and aSyn was observed, accompanied by accelerated cognitive decline, suggestive of a synergistic effect of these proteins in driving both pathology and phenotype [80]. Supporting this possible synergistic effect, aSyn knock-down in an APP transgenic mouse model of AD has been shown to reduce the degeneration of cholinergic fibers and hippocampal neurons [81]. Interestingly these effects were in the absence of any effect on either APP expression or Aβ deposition, and may point to an as yet unknown influence of aSyn on the selective vulnerability of cholinergic neurons in AD. Together these studies provide evidence supporting the provocative hypothesis that aSyn may be more than merely an idle passenger in AD pathogenesis. In contrast, some studies have suggested that aSyn may inhibit the formation of Aβ plaques, with one demonstrating a significant increase in Aβ plaque load in APP (Tg2576) mice when crossed with aSyn knockout mice [82], whilst a second study found that aSyn knockdown in APP mice increased plaque burden but decreased levels of extracellular Aβ oligomers [83]. A further study found that inoculation of APPPS1/aSyn(A30P) mice with brain homogenate derived aSyn reduced the formation of Aβ plaques and that the seeding capacity of Aβ brain homogenates was significantly reduced in the presence of aSyn [84].

Recent studies have also suggested a further, albeit indirect mechanism by which aSyn may mediate deposition of Aβ. Following the observation that exposure to recombinant aSyn leads to increased levels of Aβ in cultured PC12 cells or primary hippocampal neurons [85, 86] a recent report has suggested that aSyn may induce the production and secretion of Aβ through enhanced cleavage of APP in cultured neuronal cells [87]. It is tempting to speculate that this observation could underly the frequently observed accumulation of Aβ in synucleinopathies if so, then probing the precise mechanisms by which aSyn mediate the processing of APP could prove invaluable in the development of potentially disease-modifying therapeutics.

4) *Prion protein*

The cellular prior protein (PrP^C^) has previously been suggested as a receptor for Aβ oligomers [88, 89] although others have reported evidence to the contrary [90]. Similarly, cell surface PrP^C^ has also been described as a putative receptor that promotes the uptake of aSyn via binding to the N-terminal domain [91, 92], although others have more recently disputed these claims [89]. Thus, Aulic and colleagues reported that PrP^C^ knockdown in murine neuroblastoma cells attenuated the uptake of recombinant aSyn oligomers with a similar effect noted when comparing aSyn uptake in mouse primary hippocampal neurons prepared from wildtype or PrP^C^ knockout mice. In addition, PrP^C^ knockout mice developed lower levels of aSyn aggregates in the cortex, striatum, thalamus and hippocampus following intrastriatal inoculation with aSyn fibrils, suggesting that PrP^C^ may facilitate the uptake and aggregation of aSyn oligomers. These same authors provided evidence that the replication of scrapie prions was blocked by aSyn oligomers [93] providing a possible explanation for the observation that individuals with Creutzfeldt-Jakob disease have a more protracted disease course when there is concomitant synucleinopathy [94]. Conversely, La Vitola and colleagues found no evidence of binding between PrP^C^ and aSyn oligomers [89] and noted that both PrP^C^ knockout mice and wildtype mice were equally susceptible to aSyn oligomer-mediated toxicity and that PrP^C^ expression was not a prerequisite for these toxic effects. It remains possible that these discrepancies are a result of methodological differences between the studies. Certainly, there are many different species of aSyn oligomers, which may have a differential binding capacity with PrP^C^ and it remains possible that future studies could reveal that both PrP^C^ -dependent and PrP^C^ -independent pathways play a role in synucleinopathies [95]. On the other hand, aSyn has an unexpected role in inducing a transmissible spongiform encephalopathy with accumulation of disease-associated PrP [96]. Accordingly, aggregated aSyn is potent in cross-seeding of prion protein misfolding and aggregation in vitro, producing self-replicating states that can lead to transmissible prion diseases upon serial passaging in wild type animals.

### Anatomical sites of potential concomitant pathology in the human brain

**Fig. 3:**
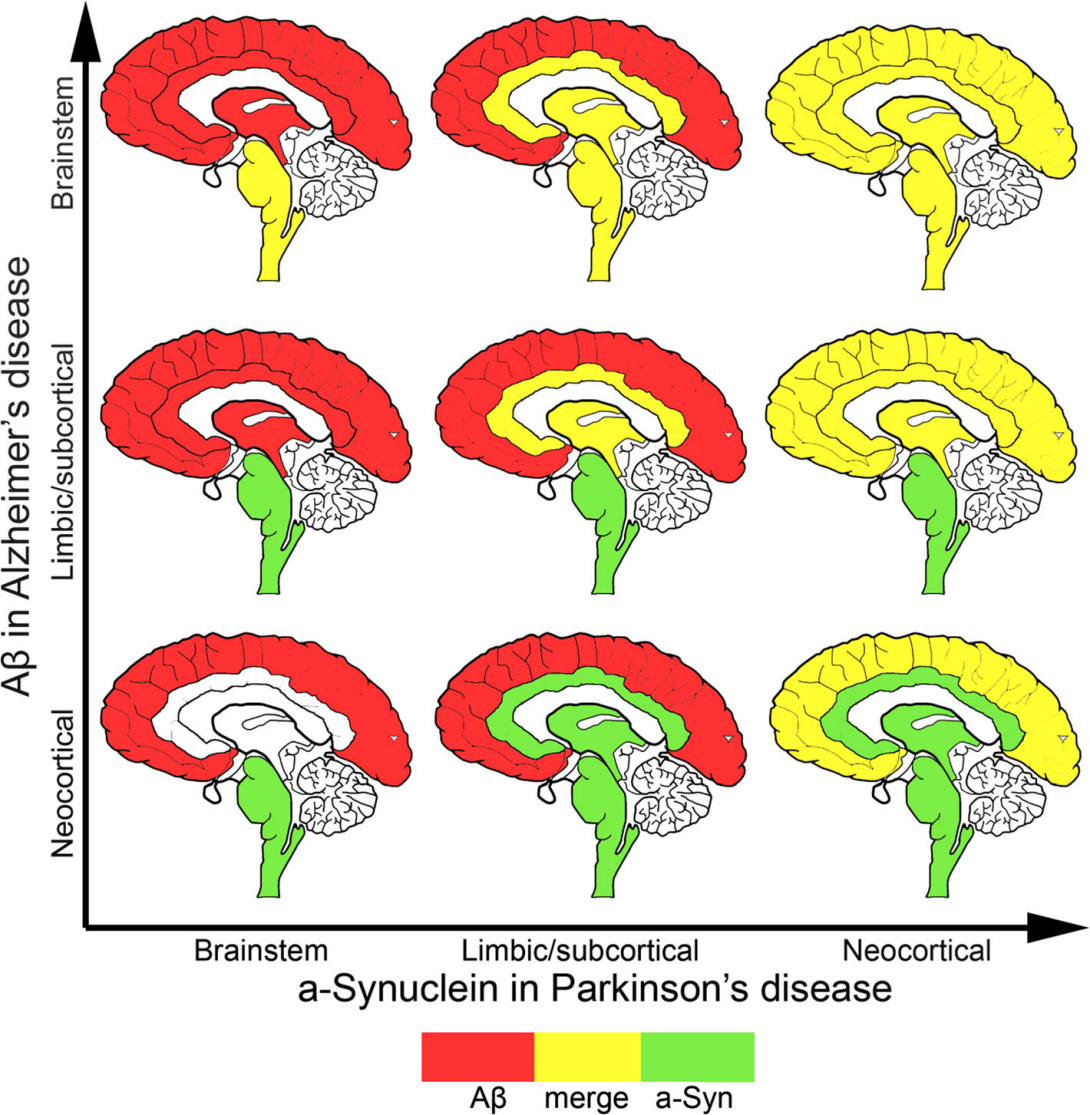
Predicted overlap (yellow) of aSyn in PD (green) with deposition patterns for amyloid-β in Alezheimer’s disease (red) in the brainstem, limbic (e.g. amygdala, hippocampus, anterior cingulate) and subcortical areas (e.g. basal ganglia) and neocortical areas. According to the Thal Phases the deposition of amyloid-β follows a neocortical to limbic/subcortical to brainstem path [102], which is opposite to that seen for aSyn according to the Braak stages of Lewy pathology [97]

**Fig. 4:**
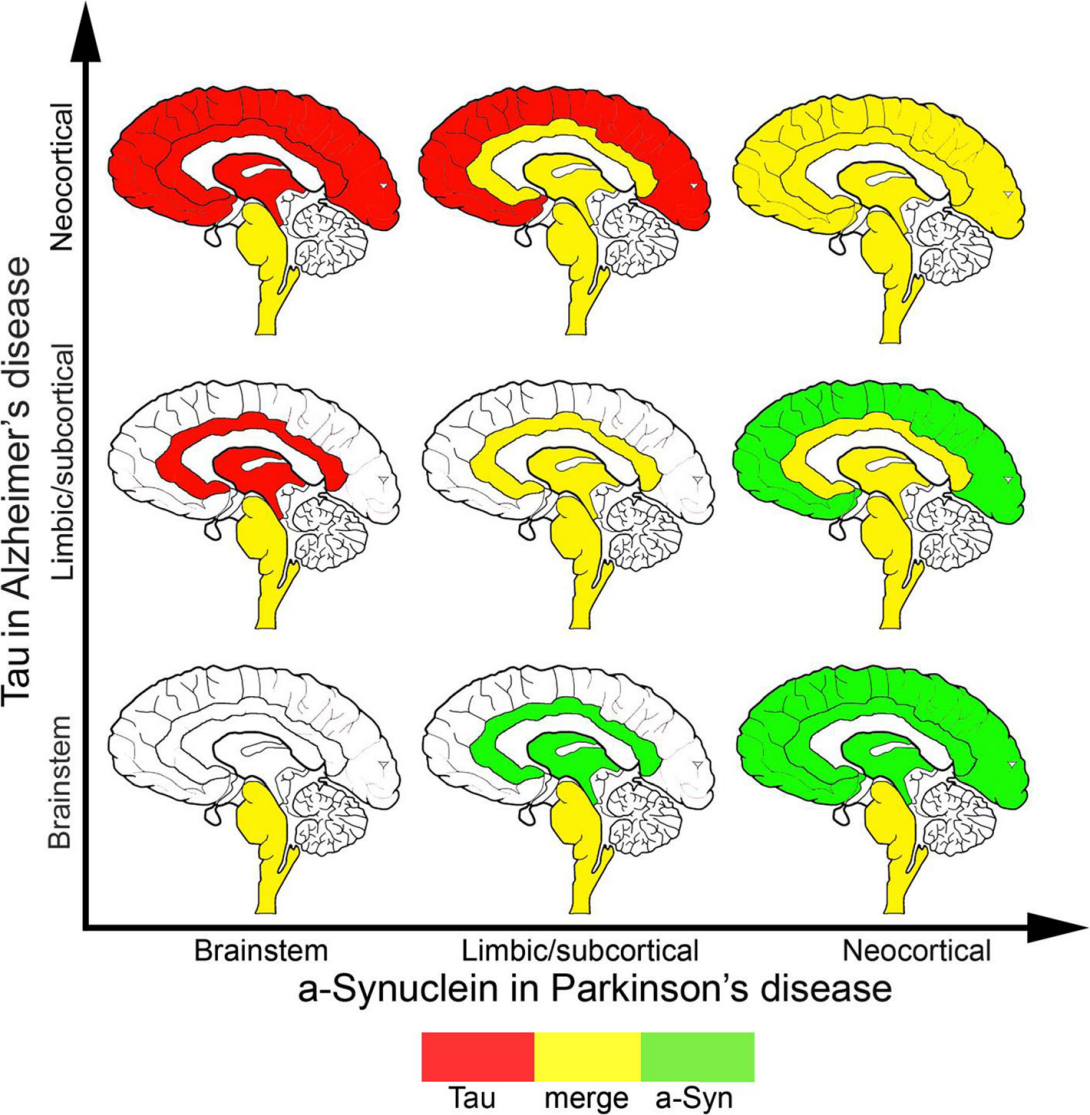
Predicted overlap (yellow) of aSyn in PD (green) with deposition patterns for tau in Alzheimer’s disease and primary age-related tauopathy (red) in the brainstem, limbic (e.g. amygdala, hippocampus, anterior cingulate) and subcortical areas (e.g. basal ganglia) and neocortical areas. According to the Braak stages of neurofibrillary tau pathology [98] the deposition of tau follows a similar involvement of anatomical systems (i.e., brainstem to limbic/subcortical to neocortex) as seen for aSyn for Lewy pathology [97]

An understanding of the typical anatomical deposition profiles of different neurodegenerative disease-associated proteins throughout the brain is vital to considering the possible impact of aSyn on the pathogenesis of other comorbid proteinopathies. As an example, Fig. 3 illustrates the predicted overlap of aSyn in PD (the most common synucleinopathy) with deposition patterns for Aβ in AD (the most common neurodegenerative proteinopathy) and Fig. 4 illustrates the predicted overlap of aSyn in PD with tau in AD/PART [97–101]. In the majority of cases, according to Braak et al. [97], in the early stages (1 to 3) of PD aSyn is predominantly deposited in the brainstem, then progresses through the limbic (including amygdala) and subcortical (including basal ganglia) regions, eventually reaching the neocortical areas in later stages of the disease. In AD, however, the pattern of Aβ deposition is essentially the opposite, with deposits first observed in the neocortex (i.e. Thal phase 1) then in the limbic and subcortical regions (i.e. Thal Phase 2 and 3) and finally being found also in the brainstem (Thal Phase 4) progressing to the cerebellum in Thal Phase 5 [102]. Thus, the opportunity for aSyn and Aβ to coexist in the same anatomical region of the brain does not present until at least one of the comorbidities is at an advanced stage. The region of the brain in which co-deposition may occur is dependent on the relative stage of each disease. Thus, an individual with early PD, but more advanced AD-related pathology would be predicted to have overlapping pathology in the brainstem only (Fig. 3). Conversely an individual with early AD, but a more advanced PD would have overlapping pathology only in the neocortex (Fig. 3). Individuals with advanced AD and PD would have the maximum potential for concomitant pathologies throughout the brain.

With respect to co-occurence of tau in AD/PART and aSyn in PD, the opportunity for the two proteins to be deposited in the same anatomical region occurs first in the brainstem, in particular the locus coeruelus, in early stages of disease, indicated as subcortical stages of neurofibrillary tangle pathology by Braak and colleagues [100] (Fig. 4). The deposition of proteins then progresses to limbic and subcortical regions. Since tau pathology (Braak stages I-IV) predominates in the limbic areas and the medial temporal lobe but not in the basal ganglia while Lewy body pathology (Braak stage 4) in the basal ganglia and limbic areas, the most likely meeting point would be the amygdala, hippocampus, entorhinal cortex and and anterior cingulate. Finally, at more advances stages the two proteins may also coexist in the neocortex (Fig. 4).

Irrespective of which proteins are co-deposited, clearly, the degree of potential overlap is dependent on the temporal stage of each disease, with fewer overlapping regions predicted early in disease and a much higher degree of potential overlap in later stages, thus emphasizing the potential contribution of comorbid proteinopathies to pathogenicity particularly in later disease. It is also important to note that these figures highlight the potential brain regions where one would predict comorbid proteinopathies to coexist, but remain agnostic to the amount of pathology present, or which if any proteinopathy is predominant. Finally, we emphasize that these images (Figs. 3 and 4) represent our knowledge based on current immunohistochemical methods to detect abnormal pathology. Hence, it remains possible that future studies mapping aberrantly deposited proteins using more sensitive and sophisticated methods, may reveal a yet unexpected and greater degree of anatomical overlap.

On an anatomical level the amygdala, a part of the limbic system located deep in the medial temporal lobe, suffers a high degree of overlapping aberrantly deposited proteins with pathology present in AD, synucleinopathies and tauopathies among others [58]. This observation has led Nelson and colleagues to recently propose that the amygdala may serve as an “incubator” for misfolded proteins and suggest that the convergence of neuropathologic comorbidities in this regions may act in synergy to drive the evolution of a pure proteinopathy to potentially more aggressive neuropathologic comorbidities [58]. This provocative hypothesis, if true, would have profound implications for both the diagnosis of neurodegenerative diseases as well as the development of novel therapeutics targeting proteinopathy. Over the next few years we hope that, using modern techniques able to probe the biochemical properties of aberrant proteins, the careful study of the common neuropathologic comorbidities, particularly in brain regions with a high degree of convergence, will provide vital information regarding the relationship between these comorbidities, the impact each may impart on the other and potentially reveal novel targets for future biomarkers and disease modifying therapies.

## Conclusions

**Fig. 5:**
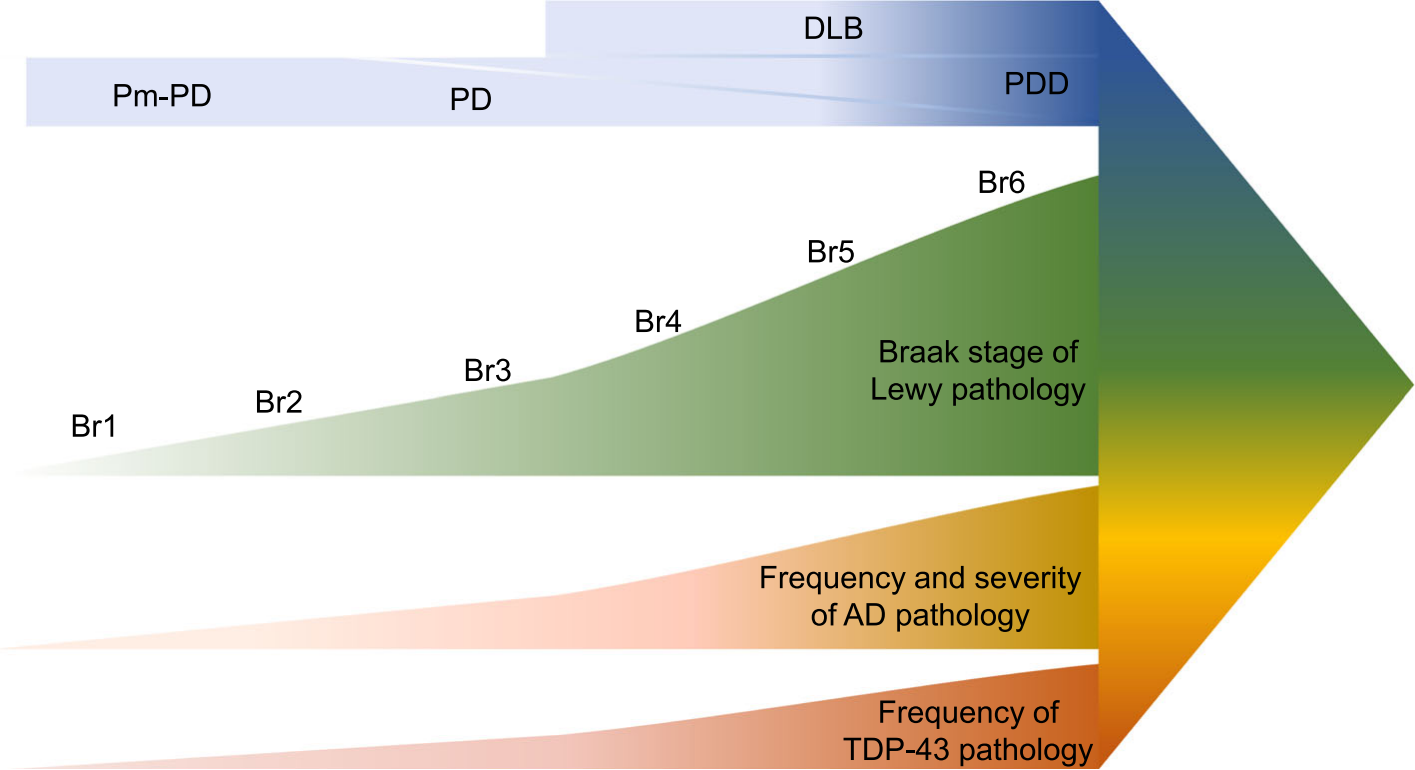
Neuropathological comorbidities associated with the clinical progression of dementia with Lewy bodies (DLB) or progression of premotor Parkinson’s disease (Pm-PD) to Parkinson’s disease (PD) and in a subset of individuals to Parkinson’s disease dementia (PDD). Clinical progression is depicted as increasing darkness of blue. Braak stage of Lewy pathology is depicted in green, frequency ad severity of Alzheimer’s disease (AD) pathology is depicted in yellow and frequency of TAR DNA-binding protein 43 (TDP-43)) pathology in orange

Given the frequency of occurrence, the impact of comorbid disease-associated proteins on disease pathogenesis in neurodegenerative proteinopathies has to date been relatively underexplored. It remains possible that the aggregation of multiple disease associated proteins in a given condition are unrelated to each other, perhaps the result of a common deleterious process in the brain, for example neuroinflammation or disturbances of the protein processing systems. However, emerging evidence suggests that the concomitant deposition of multiple disease-associated proteins may impact upon disease pathogenesis and have clinical significance. Based on neuropathological studies, as depicted in Fig. 5, the presence of comorbid proteinopathies, aSyn (depicted in green), AD-related pathology (depicted in yellow) and TDP-43 (depicted in orange), increase in tandem with each other. Thus, in premotor PD, Braak stages 1 & 2, there is minimal comorbid AD-related pathology or deposition of TDP-43. As PD progresses to a motor stage the concomitant deposition of both AD-related pathology and TDP-43 to increase in parallel, with the highest levels of comorbidity observed in PDD and DLB which encompass cognitive symptoms. Indeed, the existence of comorbid conditions often leads to a significant overlap in symptoms. For example, the clinical spectrum of parkinsonism is associated with the involvement of anatomical regions beyond those important for the organization of movement which in turn are affected by a wide variety of other neuropathologically defined disorders and pathologically altered proteins. Therefore, the clinical symptoms alone do not predict incontrovertibly the underlying molecular pathology. This circumstance jeopardizes the accurate early diagnosis of neurodegenerative proteinopathies and stratification of patients for clinical trials. Thus, even though the methods and interpretations of these aforementioned studies on comorbid proteinopathies vary considerably, on the whole these support the rationale for developing highly specific markers of disease-associated proteins involved in neurodegenerative conditions and particularly for examining all of these together in neurodegenerative conditions. Defining clusters of patients based on the patterns of comorbid proteinopathies will enhance research in disease pathogenesis, lead to an improved prognostic predictive value, and therefore, may be useful for stratifying patients for clinical trial and monitoring efficacy of novel therapies [103].

In this review we speculate that aSyn may be an as yet unrecognised potentiator of neurodegenerative diseases beyond those formally recognized as synucleinopathies. aSyn is bestowed with several characteristics that may allow this protein to impact the pathogenesis of multiple neurodegenerative proteinopathies. First, aSyn is abundantly expressed in the synapses of the brain and indeed, it is named after its localization on synaptic vesicles. All neurodegenerative diseases involve synapses (reviewed in [104]). Thus, it is very tempting to speculate that aSyn may be implicated in the synaptic dysfunction that is a feature shared by all neurodegenerative proteinopathies irrespective of the predominant pathology. Second, although disease-associated aSyn deposits are intracellular (Lewy bodies, Lewy neurites, glial cytoplasmic inclusions), aSyn and its neurotoxic oligomers are highly soluble, can be excreted into the extracellular space and are widely distributed throughout the brain [10]. This feature, (shared with tau, TDP-43 and prion protein), allows aSyn unrestricted access to both intracellular and extracellular compartments of the brain and thus permits aSyn to impart widespread effects. Third, although other neurodegenerative disease-associated proteins have been shown capable of seeing proteinopathy, it has been recently shown that aSyn may be particularly potent at cross-seeding the aggregation of other disease-associated proteins [96].

In addition to Lewy bodies or abnormally aggregated aSyn, detectable with current immunohistochemical methods, aSyn may also undergo more subtle biochemical alterations, such as phosphorylation. These changes can occur in human brains with or without neurodegenerative proteinopathies and may also precede the development of Lewy bodies [54, 105, 106]. Thus, soluble aSyn levels have been found to be approximately doubled in AD in the absence of synucleinopathy, and to more closely correlate with the degree of cognitive impairment than soluble Aβ or tau [107]. Furthermore, it is well established that aSyn can have detrimental effect on synaptic biology, in the absence of synucleinopathy [108–110]. These observations suggest that by focusing only on aggregated aSyn we may underestimate role of aSyn in comorbidities and it could perhaps be a silent potentiator driving disease. Further studies investigating the molecular and biochemical features of synaptosomal aSyn in AD, for example, have the potential to reveal that aSyn may have a more widespread role in neurodegenerative diseases beyond the synucleinopathies. If born out, this would have clear implications for the development of disease modifying therapies for neurodegenerative diseases. For example, aSyn immunotherapy could have an attenuating effect on AD-related pathology and symptoms for example. Such a hypothesis is certainly supported by the observation that co-expression of aSyn and Aβ induces a more aggressive cognitive decline in AD [80].

It is important to note, however, that the evidence linking aSyn to other neurodegenerative proteinopathies is presently largely correlative. Indeed, it remains possible that, being an aggregation prone protein, biochemical and cellular changes in other neurodegenerative proteinopathies trigger coincidental aggregation of aSyn. Thus, until such studies have been undertaken as to provide direct evidence supporting that aSyn is the driving force for other neurodegenerative proteinopathies caution should be exercised in interpreting clinicopathologic observations.

In conclusion, it is likely that future disease modifying therapies for neurodegenerative proteinopathies will entail a precision-based approach tailored to the molecular underpinning of disease in each patient. In order to achieve this goal we first need a better understanding of the interaction of different proteins in comorbid proteinopathies. This information will be vital to support effective stratification of patients for clinical trials and eventually for the successful application of disease appropriate therapeutic interventions.

## Data Availability

Not applicable.

## Abbreviations

ARTAG: ageing-related tau astrogliopathy
aSyn: alpha-synuclein
Aβ: amyloid-β
CGIs: glial cytoplasmic inclusions
DLB: dementia with Lewy bodies
FRET: Fluorescence Resonance Energy Transfer
FTLD: frontotemporal lobar degeneration
MSA: multiple system atrophy
PAF: pure autonomic failure
PART: primary age-related tauopathy
PD: Parkinson’s disease
PDD: Parkinson’s disease with dementia
PFFs: preformed fibrils
PrP^C^: cellular prior protein
TDP-43: transactive response DNA binding protein 43 kDa
